# Combating antimicrobial resistance: a shared responsibility of everyone

**DOI:** 10.1016/j.lanwpc.2024.101270

**Published:** 2024-12-23

**Authors:** The Lancet Regional Health – Western Pacific

The theme for the World Antimicrobial Resistance (AMR) Awareness Week, Nov 18–24, 2024, is “Educate. Advocate. Act now.” Despite the lack of confirmed diagnosis, some patients are being administered with antibiotics as a precautionary measure. The inappropriate use of antibiotics results in adverse health consequences in both the short term and later in life. More importantly, it poses a risk to the health of the whole population by contributing to AMR.

Antimicrobial medicines, which are the cornerstone of modern medicine, are increasingly posing a global threat as a result of their misuse and overuse in the treatment, prevention, and control of infections in humans, animals, and plants. According to the latest Global Burden of Disease data, 4.71 million deaths globally were associated with bacterial AMR in 2021, and the number could increase to 8.22 million in 2050. A variety of human activities are substantial contributors to AMR, including overprescribing of antibiotics, non-adherence, overuse of antibiotics in agriculture and aquaculture, inadequate infection control measures in health-care settings, poor hygiene and sanitation practices, and an insufficiency in new antibiotics development. Inappropriate prescription practices and misuse of antibiotics, including non-adherence and self-medication, are primary factors associated with AMR related to health-care systems.

The COVID-19 pandemic has complicated the situation of antibiotic consumption and misuse. Evidence from WHO indicates that 75% of the patients hospitalised with COVID-19 have received antibiotic treatment, despite only 8% of them having bacterial co-infections and the fact that the overall use of antibiotics did not lead to improved clinical outcomes among those without confirmed bacterial co-infections. Global antibiotic consumption soon rebounded after a decline during the pandemic, with the most pronounced increase observed in several Asia–Pacific countries, including Malaysia, Thailand, and Viet Nam. The changing patterns of infections in the post-pandemic period, characterised by the surges of non-COVID infections, higher incidence of co-infections, alongside a rise in vaccine-preventable diseases due to disruptions in routine childhood immunisation coverage during the pandemic, have resulted in heightened antibiotic use. One such example is respiratory syncytial virus (RSV), which resurged strongly in 2021 and contributed to increased antibiotic use, with a third of paediatric patients with RSV receiving unnecessary antibiotics.

Inappropriate antibiotic prescribing is not solely the responsibility of prescribers. Various determinants across several domains also influence these decisions. These determinants include patient–doctor interactions (eg, poor communication skills, time constraints, and high demand for antibiotics from patients), primary care settings (eg, limited capacity of health-care professionals and unviability of supporting tests and guidelines), and the nationwide health-care system (eg, the health-care payment model and regulations). In medical care systems, the primary goal often centres on using antibiotics to address immediate health conditions, while the potential consequences of the misuse of antibiotics on AMR might not always be adequately considered during the treatment process. AMR is quite often perceived as invisible to the general public—mortality or morbidity associated with AMR might be interpretated as outcomes attributed to the underlying disease rather than to AMR and repercussions might manifest over the long term or affect others, including future generations. Meanwhile, misconceptions on the use of antibiotics are prevalent, including the belief that antibiotics are effective against colds. This underscores the urgent need to enhance universal awareness of AMR and to implement effective strategies for guiding rational antibiotic use from multiple sectors.

AMR is a shared responsibility. In 2022, Bangladesh launched the antibiotic packaging initiative, printing red markings and the text “antibiotic” on the primary and secondary packaging, as well as the phrase “Do not take antibiotics without the prescription of a registered physician.” As of January, 2024, 80% of pharmaceutical companies in Bangladesh had adopted this new packaging standard. Clinical and patient-centred interventions are also showing promising results. A recent cluster randomised controlled trial indicates that an intervention package consisting of clinician training, a peer support mechanism, computerised decision aid, public commitment letter, and patient leaflet was effective in reducing antibiotic use related to respiratory tract infections in rural China. The long-term effects of these initiatives are yet to be measured, but they serve as insightful examples for making antibiotics and AMR more visible and presenting cost-effective strategies to increase awareness among the public, health-care professionals, and retailers, which might potentially modify their behaviours. Nevertheless, more needs to be done. Although national action plans on AMR in southeast Asia have underpinned the need for monitoring knowledge, attitude, and practices among professionals and the public, only two counties (Indonesia and Myanmar) mentioned evidence-based behaviour change campaigns being planned. Sustained efforts should be directed towards the development and evaluation of concrete behavioural change campaigns. WHO has identified better individual optimal antibiotic use among patients as a key policy target to reduce AMR. As individuals, we can start by understanding antibiotics and AMR, improving personal hygiene and sanitation, receiving regular vaccinations to reduce the risk of infections and thereby the use of antibiotics, and always seeking medical consultation when using antibiotics.

Empowering the public and health-care professionals with comprehensive knowledge on antibiotic use and AMR is the first step to change behaviours. However, we must also recognise the significance of exploring additional interventions beyond education to effectively combat AMR. Overall, AMR is a profound challenge that calls for the commitment and engagement of not just our health-care systems, but also regulatory authorities, pharmacies, patients, and every member of our communities to promote the responsible use of antibiotics.

